# An Outcome-Weighted Network Model for Characterizing Collaboration

**DOI:** 10.1371/journal.pone.0163861

**Published:** 2016-10-05

**Authors:** Matthew B. Carson, Denise M. Scholtens, Conor N. Frailey, Stephanie J. Gravenor, Gayle E. Kricke, Nicholas D. Soulakis

**Affiliations:** 1 Department of Preventive Medicine, Feinberg School of Medicine, Northwestern University, Chicago, IL, United States of America; 2 Department of Emergency Medicine, Feinberg School of Medicine, Northwestern University, Chicago, IL, United States of America; Tianjin University, CHINA

## Abstract

Shared patient encounters form the basis of collaborative relationships, which are crucial to the success of complex and interdisciplinary teamwork in healthcare. Quantifying the strength of these relationships using shared risk-adjusted patient outcomes provides insight into interactions that occur between healthcare providers. We developed the *Shared Positive Outcome Ratio* (SPOR), a novel parameter that quantifies the concentration of positive outcomes between a pair of healthcare providers over a set of shared patient encounters. We constructed a collaboration network using hospital emergency department patient data from electronic health records (EHRs) over a three-year period. Based on an outcome indicating patient satisfaction, we used this network to assess pairwise collaboration and evaluate the SPOR. By comparing this network of 574 providers and 5,615 relationships to a set of networks based on randomized outcomes, we identified 295 (5.2%) pairwise collaborations having significantly higher patient satisfaction rates. Our results show extreme high- and low-scoring relationships over a set of shared patient encounters and quantify high variability in collaboration between providers. We identified 29 top performers in terms of patient satisfaction. Providers in the high-scoring group had both a greater average number of associated encounters and a higher percentage of total encounters with positive outcomes than those in the low-scoring group, implying that more experienced individuals may be able to collaborate more successfully. Our study shows that a healthcare collaboration network can be structurally evaluated to characterize the collaborative interactions that occur between healthcare providers in a hospital setting.

## Introduction

Federal agencies such as the Centers for Medicare and Medicaid Services (CMS) and the Agency for Healthcare Research and Quality (AHRQ) are working to promote care coordination across the nation in order to improve healthcare quality [1]. While there is uncertainty about the definition and proper measurement of care coordination quality [2], collaboration among a patient’s numerous providers is fundamental to its success. An NIH-funded report defines communication and collaboration between providers within and across institutions as the two prerequisites to care coordination and concludes that effective collaboration improves care coordination [3]. In the past, quality measures were developed to assess physician-nurse and physician-pharmacist collaborations, and to evaluate the effectiveness of the programs designed to foster collaboration [2]. The current practice for clinical quality measure development involves gathering a consensus from the scientific literature, clinical practice guidelines, and domain experts [4, 5]. Implementing these approaches can be effective on the patient level, but do not reveal a comprehensive picture of care collaboration in a healthcare facility.

Research involving complex networks [6–14] has surged since the turn of the century and spanned a variety of disciplines including sociology [15, 16], biology [17–19], medicine [20–22], chemical engineering [23–26], material science [27], finance [28], and others [29]. The affiliation network, a commonly studied type of social network, is a useful structure for identifying relationships between individuals based on shared events or interests. These networks often consist of two disjoint sets of entities, “actors” and “groups”, with a link indicating that an actor is associated with a group. From this bipartite network one can create a projection consisting of actor nodes with links between them if they have co-membership to one or more groups. Research with affiliation networks often focuses on co-authorship [30] and collaboration [29, 31, 32].

A collaboration network can help to characterize the relationships formed between thousands of providers caring for shared patients in a hospital setting. However, storing and modeling the underlying data to facilitate network generation can be a challenge. Because the components of a healthcare event are highly interconnected, a traditional relational data model does not scale well as the data set size increases [33]. A graph data model, however, offers three advantages for representing large, highly connected data sets. First, the nature of the model is such that all entities are logically linked through a variety of relationships. This inherent connectedness of all data results in faster query times compared to a relational model, which requires that tables be joined to create relationships between entities, a process that quickly increases in computational time as the number of joins are increased [34]. Second, the lack of a predefined schema makes the graph data model flexible; updates and modifications do not require table alterations. Third, the labeled property graph model, the most common variation of graph data model, allows properties to be added to both nodes and relationships. Nodes can also contain one or more labels. These features allow for a rich representation of the data and facilitate ad hoc querying of the associated database [33, 34].

For example, consider a patient, a provider, an encounter, and activities occurring during the encounter as four components of a healthcare event. The logical relationship between these components could be defined as follows: “Provider *P* performs Activity *A* for Patient *X* during Encounter *E*”. Each provider may perform one or more activities during each encounter and may be involved in multiple encounters per day. The relationship between a specific instance of an activity, a provider, and an encounter can be modeled using a hyperedge [29, 35]. In this case, each hyperedge would describe the following: “Provider *P* performed Activity *A* during Encounter *E*”. Not only does this construct identify a relationship between the three entities, but it also provides data flexibility by enabling extraction of activity subsets and associated providers. This identification is especially useful when examination of a specific activity group within a protocol is warranted, e.g., an analysis of all providers and activities associated with patient discharge.

A number of previous studies have analyzed the professional networks of providers within healthcare systems [36–49]. A few studies have focused on identifying targets for improving care coordination on both organizational and patient levels [37, 40, 42, 50]. However, each of these studies is based on either a survey or direct observation [51]. Most include small patient and provider samples, which may not capture larger trends in the healthcare system of interest. Some studies have made use of larger data sets but focused specifically on interactions between physicians and other physicians or nurses and other nurses [38, 44–48, 52]. In addition, the majority use single-source commercial or Medicare claims data [43, 44, 47, 52–54].

CMS recognizes the importance of the electronic health record (EHR) as a rich resource for gathering information and promoting coordination of care. Incentive programs are offered to encourage Meaningful Use of EHRs [2]. Though it can be difficult to use EHR data to construct an interaction map of providers and the patients they share [55], there are precedents in the literature [38, 56, 57]. Recently, we showed that EHRs can be used to effectively identify providers affiliated with a set of common heart failure patients and subsequently demonstrated methods for visualizing and describing collaborative interactions among these providers [58]. We now extend these methods by developing an informative edge-weighting scheme, which allows for data-driven measurements of the relationships in a collaboration network [59]. To monitor and improve healthcare quality, a weight must impart actionable information about the patients and providers involved.

In this study we propose a method to construct a collaboration network from electronic health record (EHR) data and establish a generalizable, graph-based framework for calculating and measuring the *Shared Positive Outcome Ratio (SPOR)*, an objective composite measure that quantifies the concentration of risk-adjusted positive outcomes for each pair of actors over a set of shared events. Our objective here is to use this flexible model to characterize pairwise collaboration in terms of patient encounter outcomes, given the frequency of collaboration between providers. Our long-term goal is to understand, monitor, and improve provider collaboration by creating a highly adaptable, scalable, network-based platform that measures differences in working relationships between various providers.

## Materials and Methods

### Quantifying and Measuring Collaboration: the Shared Positive Outcome Ratio (SPOR)

The SPOR is a pairwise metric that quantifies the ratio of risk-adjusted positive outcomes shared between two providers vs. risk-adjusted positive outcomes shared with other providers. We describe the risk adjustment process and subsequently explain the metric development below. While our methods are described using the terms “provider” and “encounter” as appropriate for our application, we note that the methodology can be adapted to other sets of actors and events.

#### Calculating Risk-adjusted Outcomes

A simple binary scheme for capturing positive (1) and negative (0) outcomes for each encounter would fail to capture the baseline probability of a positive outcome for the patient involved in the encounter. This might unduly penalize a provider pair for negative outcomes in patients with very low chance of a positive outcome at baseline, or unduly reward a provider pair for positive outcomes in patients with a high chance of a positive outcome at baseline. For this reason, we propose using “risk-adjusted outcomes” that range from 0 to 1 and are: (a) higher for positive outcomes for encounters with patients that had lower positive outcome probabilities, and (b) lower for positive outcomes for encounters with patients that had higher positive outcome probabilities, regardless of their experience with providers.

Our method to calculate “risk-adjusted” outcomes incorporates logistic regression modeling for a positive outcome for the encounter and a set of baseline covariates. Let *I* be the set of encounters. Given an encounter *i* ∈ *I*, the outcome related to this encounter, *y*_*i*_, can be defined as

$$y_{i}=\{\begin{array}{c} \;1\to positive\;outcome\\ \;0\to negative\;outcome \end{array}$$ (1)

For a given set of baseline covariates, logistic regression can be used to model associations of the baseline values with the outcomes and positive outcome probabilities can then be estimated for each encounter. If *x*_1*i*_,*x*_2*i*_,…,*x*_*ri*_ are values of *r* baseline covariates for the patient involved in encounter *i*, let

$$p_{i}=\mathrm{Pr}\left(y_{i}=1\right)=\frac{e^{\beta_{0}+\beta_{1}x_{1i}+\beta_{2}x_{2i}+\ldots +\beta_{r}x_{ri}}}{1+e^{\beta_{0}+\beta_{1}x_{1i}+\beta_{2}x_{2i}+\ldots +\beta_{r}x_{ri}}}$$ (2)

where the β parameters are estimated using logistic regression. We then define a risk-adjusted outcome, *r*_*i*_, as follows:

$$r_{i}\left(y_{i}\right)=\frac{1+\left(y_{i}-p_{i}\right)}{2}$$ (3)

Note the nature of the values of *r*_*i*_ given values of *y*_*i*_ and *p*_*i*_:

$$y_{i}=\{\begin{array}{l} \;1,\;high\;P_{i}\to r_{i}\;is\;close\;to\;0.5\\ \;0,\;high\;P_{i}\to r_{i}\;is\;close\;to\;0\\ \;1,\;low\;P_{i}\to r_{i}\;is\;close\;to\;1\\ \;0,\;low\;P_{i}\to r_{i}\;is\;close\;to\;0.5 \end{array}$$ (4)

This has the effect of generously rewarding unexpectedly good outcomes (*r*_*i*_ close to 1) and heavily penalizing unexpectedly bad outcomes (*r*_*i*_ close to 0) while giving smaller rewards and penalties for expected outcomes (both close to 0.5). The intent of this risk adjustment is to account for variability in the characteristics of shared encounters between providers and should be informed by domain experts when the SPOR model is applied.

#### Deriving the SPOR

Let *J* be the set of providers and consider a subset of provider pairs (*j*,*j*′) such that $A_{j}\cap A_{j^{\prime }}\neq 0$, where *A*_*j*_ is the set of encounters in *I* involving *j*.

The SPOR is a ratio of two indices. The first, which we call the *shared encounter index* (SEI), is intended to measure the frequency two providers are involved in the same encounters independent of outcome. Modeled after the Jaccard index [60], this statistic can be defined as:

$$SEI_{j,j^{\prime }}=\frac{\left|A_{j}\cap A_{j^{\prime }}\right|}{\left|A_{j}\cup A_{j^{\prime }}\right|}$$ (5)

where *A*_*j*_ and *A*_*j’*_ are the sets of patient encounters involving providers *j* and *j’*, respectively. The second, termed the *shared positive outcome index* (SPOI), measures the ratio of outcomes for encounters shared by two providers relative to outcomes for all encounters involving either provider. The SPOI is defined as:

$$SPOI_{j,j^{\prime }}=\frac{\sum_{A_{j}\cap A_{j^{\prime }}}r_{i}\left(y_{i}\right)}{\sum_{A_{j}\cup A_{j^{\prime }}}r_{i}\left(y_{i}\right)}$$ (6)

The ratio of the SPOI and the SEI summarizes the observed risk-adjusted outcomes for encounters shared by two providers, relative to the expected outcomes:

$$SPOR_{j,j^{\prime }}=\frac{SPOI_{j,j^{\prime }}}{SEI_{j,j^{\prime }}}$$ (7)

This pairwise measurement of the strength of an encounter-sharing relationship attempts to answer the following question for any pair of providers: *How many more good outcomes do these two providers achieve when they work together versus when they work with other providers*? For each provider, the “concentration” of good outcomes with each collaborator is measured. See Fig A and Table A in S1 File for examples of the relationship between patterns of shared patients and resulting SPOR values.

### Testing the Method with Clinical Data

#### Data Processing Overview

We implemented a multi-step process to generate the data set with which we tested our method. First, we built a graph data model to represent providers, activities, and associated encounters. Second, we extracted patient encounter data from electronic health records. For each encounter, we identified and extracted the set of all activities, a list of all healthcare providers who performed these activities, and a list of attributes associated with each entity type. In addition, we identified an outcome and an acuity measure that we used for risk-adjustment modeling of the encounters. In this study, “acuity” is equivalent to the ESI-level (Emergency Severity Index) (http://www.esitriage.org). After acquiring the initial data set, we processed and organized the raw data by checking for consistency and logic, dealing with missing values (e.g., IDs, position titles, activities, etc.), and omitting patient encounter records with inconsistencies. Using the parameters defined in our graph data model, we loaded the cleaned set into a graph database, which served as a data repository and query engine for our analysis. Next, we created a provider-encounter network to identify providers who shared encounters. From this bipartite network we created one-mode projections, termed provider collaboration networks. We calculated the SPOR for each pairwise relationship and set this SPOR as the edge weight. Finally, we performed statistical analysis on our networks.

#### Software Used

**Fig 1:**
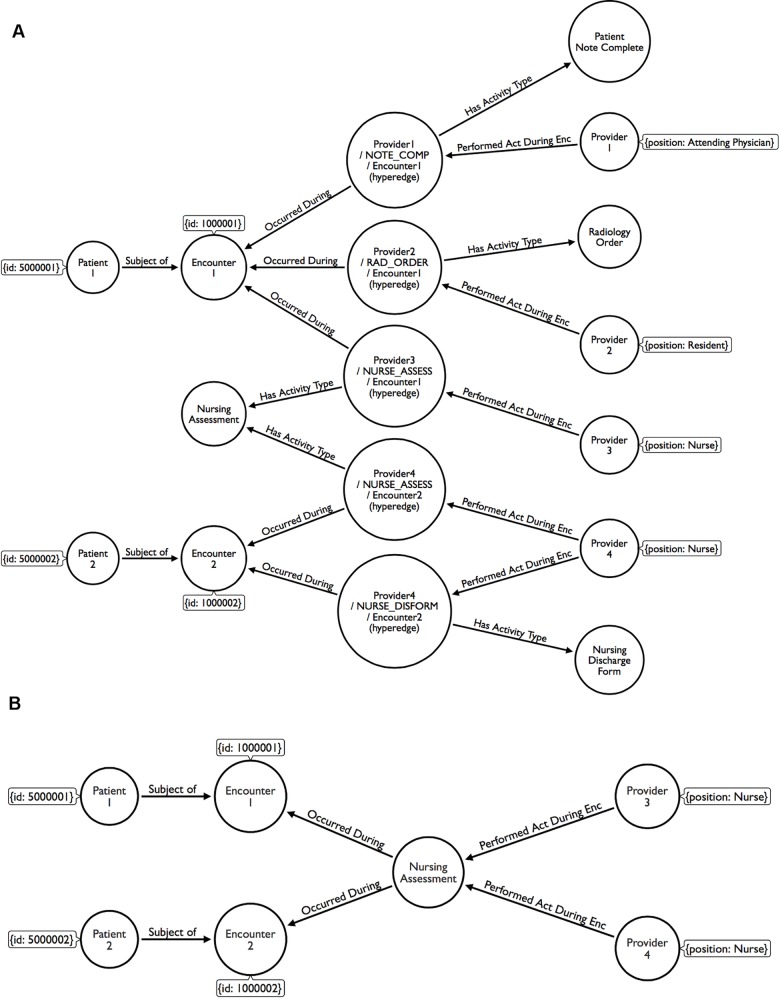
A simple example of the graph data model showing five actions performed by four providers during two encounters. (A) Providers 1, 2, and 3 each performed one activity during encounter 1, while provider 4 performed two activities during encounter 2. A hyperedge was used to represent an instance of activity during an encounter. Notice that both Provider 3 and Provider 4 performed a “Nursing Assessment” activity during different encounters. (B) Without hyperedges between the provider and the encounter nodes, it would not be possible to determine, for example, which provider performed the “Nursing Assessment" during encounter 2.

Patient encounter, provider, and activity data was extracted from Cerner’s FirstNet**®** software, an EHR system used by the emergency department, via extract, transform, and load (ETL) scripts and stored in operational data stores (ODSs) by the Northwestern Medicine Enterprise Data Warehouse (NM EDW), a repository for electronic health record data. T-SQL queries from Microsoft SQL Server Management Studio [61] were used to export the raw data set to comma separated value (.csv) files. We used the Neo4j graph database management system [35] to create the data repository for our analysis and Cypher (Neo4j’s native query language) to query the database. Data was accessed and extracted from the database using Python [62] and the Py2neo library [63]. We used Python’s NetworkX package [64] to create and analyze the networks along with custom functions to calculate the SPOR. Network visualization, statistics, and community detection were performed using Gephi [65, 66]. R [67] was used to perform statistical analysis and calculate risk-adjustment factors. Cypher was used to update the graph database with data generated by our analyses. The graph data model example in Fig 1 was created using Arrows [68].

#### The Graph Data Model

We identified the actions performed by each provider during each encounter by linking patient data elements stored in the EHR to each activity type. Once this information was gathered, we translated it into a labeled property graph model. A simplified example of the graph model for two encounters and four providers is shown in Fig 1. Hyperedges were used to identify instances of provider activities. As mentioned previously, this method allowed us to identify the relationships between a provider, an activity, and an encounter. Hyperedges also enabled data subsetting and filtering. For example, the SPOR metric could be calculated for providers based only on clinical actions (leaving out administrative activity). In this study, we do not distinguish between or add weight to any specific activity or group of activities. Therefore, all involved providers are considered equal contributors to an encounter outcome in the calculation of the SPOR.

#### Data Set

The Emergency Department (ED) of Northwestern Memorial Hospital (NMH) is a large, urban, academic medical facility with an annual volume of over 86,000 patients in 2014 [69]. We retrospectively acquired information from a subset of the electronic health records for patients who were admitted to the ED between January 1, 2012 and December 31, 2014 using the NM EDW. The primary outcome of interest was the likelihood to recommend (LTR) as reported by the Press Ganey Associates, Inc. Patient Satisfaction Survey, which is the most widely used commercial patient satisfaction instrument in the United States. The survey process for each patient was conducted as follows. First, the hospital sent patient encounter information to Press Ganey three days after the patient was discharged from the ED. Press Ganey then directly sent the patient a survey by email or mail depending on the preferred method of contact within 1 day. The patient completed the survey and returned it to the hospital, the average return time being 7 days and 2 weeks for surveys returned by email and mail, respectively. The survey return for the period corresponding to our data set was approximately 12%. A patient’s likelihood to recommend NMH’s ER (LTR) was measured on a 5-point Likert scale; LTR+ = score of 5/5, or highly likely to recommend; LTR- = score of 4/5 or below, not highly likely to recommend. The final cleaned set included 6,822 ED encounters of which 4,120 were LTR+ and 2,702 were LTR-. This set included 2,743 providers, each belonging to one of seven general categories (physician, resident, student, nurse, pharmacist, or other) and holding one of 103 positions. We identified three general activity categories (orders, notes, intake-output), which include 24 subcategories. Each activity had one of 18 action types including order, complete, performed, verified, modified, discontinued, and others. Each provider performed one or more activities during each encounter, with each instance counted as a separate event. All included provider actions occurred as part of the ED encounter. Northwestern University’s Institutional Review Board approved the study with a waiver of patients’ informed consent.

#### Evaluating Provider Collaboration

**Fig 2:**
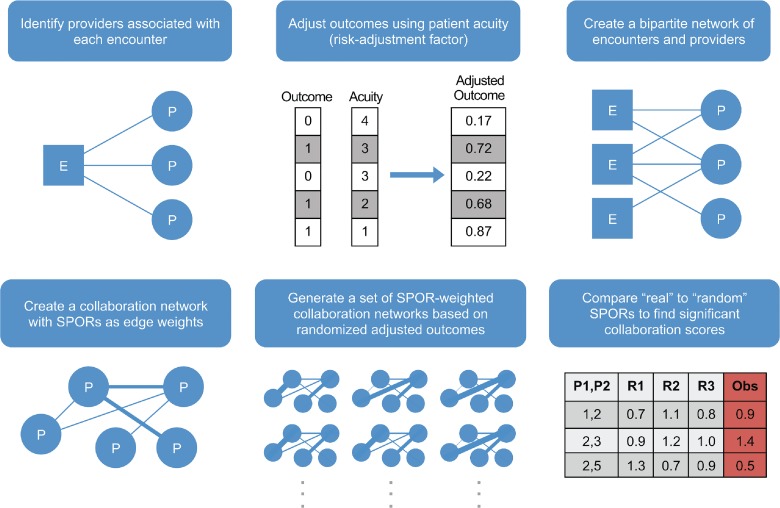
The collaboration evaluation strategy used in this study.

Using the theoretical framework defined above, we measured and evaluated collaboration for a group of providers over a set of encounters. The evaluation process is summarized in Fig 2. Our first step was to extract providers, associated encounters, and properties of each from the graph database. Next, we calculated risk-adjusted outcomes using a logistic regression model. We then created a bipartite provider-encounter network with the risk-adjusted outcome as an encounter property. From this we created a provider collaboration network, calculated the SPOR for each relationship, and added the SPOR as an edge weight. The number of shared encounters between two providers was also stored as an edge property. During this process, the resulting collaboration network was adjusted to include only those relationships between providers that exceeded a given threshold number of encounters (between two and ten in this study). Subsequently, we created an adjacency matrix for this network in which each element corresponded to the SPOR of the collaborative relationship between two providers. We then returned to the provider-encounter network and randomly permuted the risk-adjusted outcomes for each encounter. Following the previous procedure, we created 1,000 “random” collaboration networks followed by an adjacency matrix for each populated with SPOR values. We compared each SPOR in the real network to SPORs for corresponding edges in each random network. We then calculated a p-value for each pair of providers by identifying the number of times the SPOR value for that pair in a random network exceeded the SPOR value for that pair in the real network (see Table B in S1 File for an example). We classified those collaborations with a p-value ≤ 0.05 as “high-scoring” and p-value ≥ 0.95 as “low-scoring”. For the high and low SPOR groups, we identified the number of times each provider was involved in these collaborations. Next, we collected those providers for whom at least 5% of their total collaborative interactions were included in the high- or low-scoring groups.

The SPOR value for a collaborative relationship was highly volatile for those providers sharing few patients. When providers share only a small number of patients, one high- or low-scoring relationship has a large impact on the SPOR value. Because of this effect, we built a series of collaboration networks using an increasing threshold for the minimum number of shared patients required to constitute a collaborative relationship between a pair of providers and subsequently examined the distribution of SPORs across each network. This helped identify the threshold value that would generate a stable distribution and ensured that the score for each collaborative relationship was dependent on a minimum number of events, which made subsequent analysis more reliable.

## Results

### Collaboration Network

**Fig 3:**
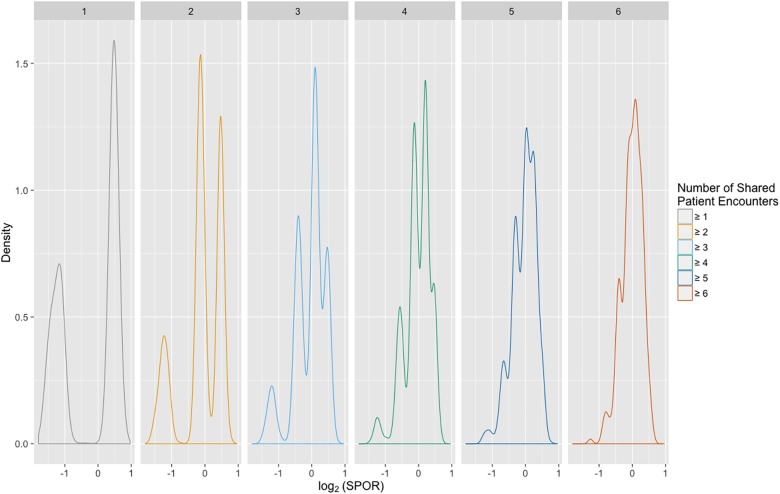
SPOR value distributions. The distribution of SPOR values across the provider collaboration network as the number of collaborations required between providers for inclusion into the network was increased. The distribution stabilized when the lower limit on shared encounters was set at six. The x-axis value shown is on a log_2_ scale, which means –1 is equivalent to a SPOR of 0.5 and a value of 1 is equivalent to a SPOR of 2, while the expected SPOR value is 0.

We created a set of collaboration networks, each with a different threshold on the number of shared encounters required to connect a pair of providers. We found that the distribution of SPOR values moved closer to normal as the threshold was increased (Fig 3). We identified extreme SPOR values for four of these collaboration networks with the shared encounter threshold at ≥ 2, ≥ 4, ≥ 6, ≥ 8, and ≥ 10. Table 1 and Fig B in S1 File provide summary statistics for these networks including the percentage of collaborative relationships in each network with high and low SPOR scores. As the required number of shared encounters between providers was increased, the 90^th^ and 95^th^ percentiles slowly decreased, while the 5^th^ and 10^th^ percentiles slowly increased. The mean remained approximately constant, while the standard deviation decreased. This highlights the volatility in the low-threshold networks and demonstrates the need to set a minimum requirement on the number of shared encounters. Based on the SPOR value distributions, we chose a threshold of ≥ 6 shared encounters between a pair of providers to define collaboration, which removed the noise of nascent relationships. We performed all further analysis on this network. Because this threshold is context- and data-dependent, this process should be performed separately for each data set included in a study.

**Table 1:** Statistics for five provider collaboration networks based on an increasing number of shared patients required for a collaborative interaction.

	Collaboration is defined as a working relationship between two providers who share. . .				
	≥ 2 encounters	≥ 4 encounters	≥ 6 encounters	≥ 8 encounters	≥ 10 encounters
**# providers**	1,479	769	576	456	359
**# collaborations**	54,030	14,742	5,615	2,534	1,327
**# SPORs p-val** ≤ **0.05**	2,880 (5.3%)	778 (5.3%)	295 (5.2%)	158 (6.2%)	63 (4.7%)
**# SPORs p-val** ≥ **0.95**	2,793 (5.2%)	790 (5.4%)	336 (5.9%)	147 (5.8%)	91 (6.9%)
** **			**SPOR Values **		
**95th %**	1.44	1.35	1.28	1.25	1.22
**90th %**	1.39	1.27	1.22	1.20	1.18
**Mean**	0.99	0.99	1.00	1.00	1.00
**10th %**	0.47	0.70	0.76	0.81	0.82
**5th %**	0.41	0.63	0.70	0.74	0.75
**SD**	0.30	0.21	0.18	0.15	0.14

As the shared patient threshold increased, the mean SPOR remained constant. The 5^th^ and 10^th^ percentiles increased as the threshold increased, while the 90^th^ and 95^th^ percentiles decreased. The standard deviation decreased as well, indicating that the SPOR distribution was more stable for networks with a higher threshold.

The collaboration network (≥ 6 encounters, see Fig C in S1 File) included 574 providers and 5,615 relationships. The network diameter was 9 and the network density was 0.034, a value that highlights the fact that a large proportion of the providers in the network have not collaborated with each other. The average degree, i.e., the average number of collaborations for an individual provider, was 19.5. The network modularity was 0.228, suggesting that, while there were groups of providers who worked together more frequently compared to others, these groups had significant overlap. The average clustering coefficient, 0.586, indicated a moderate tendency for a collaborative pair of providers to have other collaborators in common. The average path length was 2.4, meaning that the average provider was 2–3 steps removed from a collaboration with any other provider.

To examine high- and low-scoring collaborative relationships more closely, we identified the providers involved with the largest number of these interactions. Specifically, we chose those providers for which these high- or low-scoring collaborative interactions represented at least 5% of their total collaborative interactions in the network. Using this definition, we identified 29 providers with multiple high-scoring collaborations, indicating potential top performers in terms of patient satisfaction (Table C in S1 File). We found 38 providers with multiple low-scoring collaborations (Table D in S1 File). Fifteen providers belonged to both the high- and low-scoring groups. Interestingly, while the average number of high- or low-scoringcollaborative relationships was similar across the two groups (8.03 and 8.26, respectively), the average number of total collaborations for the providers in the high-scoring group was greater. Providers belonging to the high-scoring group had a weighted average of 6.6% of their total collaborations identified as significantly high scoring, while the low-scoring group had a weighted average of 8.3% of their total collaborations identified as significantly low scoring. When considering only those providers unique to the scoring groups (white rows in the Tables C and D in S1 File), the weighted averages were 9% and 13% for the high- and low-scoring groups, respectively. Providers in the high-scoring group had both a higher average number of associated encounters (255 vs. 220) and a higher percentage of total encounters with positive outcomes (61% vs. 57%) than those in the low-scoring group. These could be indications that as providers gain more experience, their ability to form successful collaborations with other providers increases.

**Fig 4:**
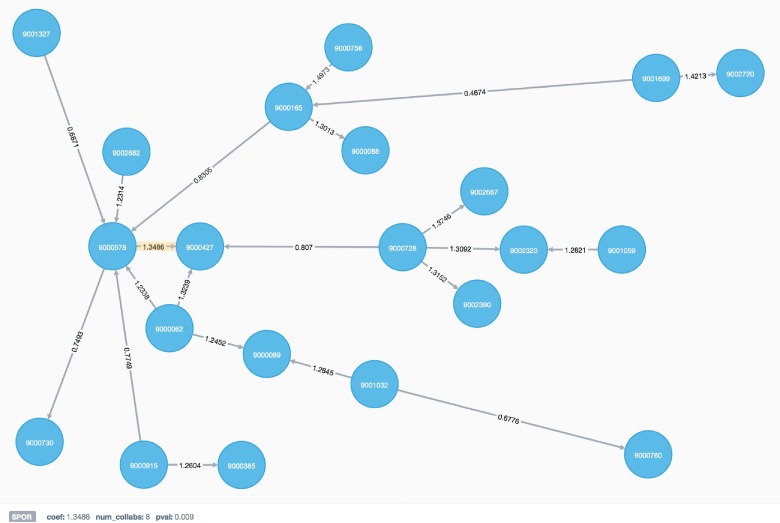
An example provider collaboration network showing 21 providers and 21 SPOR relationships. Properties associated with the highlighted edge (yellow) including the SPOR coefficient, the number of shared patient encounters between the two providers (num_collabs), and an indication of the significance of the SPOR coefficient (p-value) are shown in the bottom left. The proximity of nodes to each other is based on the SPOR coefficient, with high-scoring relationships being shorter in length than low-scoring relationships.

Fig 4 shows an example collaboration network from our graph database. Twenty-one providers are shown as blue nodes labeled by a randomized ID number. The 21 relationships shown between the providers are labeled with the SPOR value for the respective collaboration. One relationship is highlighted in yellow with associated properties including the SPOR value and the total number of encounters shared between the pair of providers displayed below.

### Descriptive Statistics for Test Data

Table 2 provides a summary of encounter-level statistics. We found that the average emergency department patient stayed approximately 4.5 hours and was given acuity level 3 (“Urgent”). An average visit involved nine providers, each of whom performed two to three activities. The mean values of length of stay and number of providers generally increased with acuity level from category 5 (Non-urgent) to category 1 (Resuscitation) (see Table E in S1 File). Our data set contained 4,477 female and 2,345 male patients. The mean (median) age for females was 50 (51). For males, mean (median) age was 53 (54) (see Fig D in S1 File).

**Table 2:** Encounter-level Statistics for the Test Data Set.

	Provider Count	LoS (hrs.)	Activity Count	Action Count
**Minimum**	1.0	0.2	2.0	2.0
**1**^**st**^ **Quart**	6.0	2.7	9.0	14.0
**Median**	8.0	4.0	15.0	23.0
**Mean**	9.2	4.5	16.4	25.9
**3**^**rd**^ **Quart**	11.0	5.7	22.0	35.0
**Max**	39.0	51.9	102.0	152.0
**St. Dev.**	4.3	2.8	8.6	15.3

A summary of encounter-level descriptive statistics showing the number of providers who performed at least one activity during the encounter, length of stay (LoS) in hours, activity count (the number of times an activity type occurred), and action count (the number of activity instances or provider actions).

The provider-encounter network consisted of 2,743 providers and 6,822 encounters for a total of 9,565 nodes. The network contained 59,265 directed edges, where each directed edge from a provider to an encounter indicated that the provider performed at least one action during the encounter. The network density was 0.002, the average out-degree (the average number of provider-associated encounters) was 21.6, and the average in-degree (the average number of providers associated with an encounter) was 9.2.

## Discussion

We have proposed a method to construct a collaboration network using electronic health record (EHR) data and to evaluate pairwise relationships with a novel metric, the Shared Positive Outcome Ratio (SPOR). Using our method and an emergency department test data set, we have shown that, in terms of patient satisfaction, collaborative relationships between pairs of providers are not equal, and that it is possible to identify extreme high- and low-scoring relationships over a set of shared patient encounters. On a global level, collaboration between providers appears to be highly variable in terms of patient satisfaction. In addition, a majority of providers are involved in both high- and low-scoring relationships, suggesting that collaboration is also highly variable on an individual level. Our results indicate that an increase in the number of shared encounters between a pair of providers may improve collaboration in some cases. Increasing the threshold for the minimum number of shared patient encounters between a pair of providers in the collaboration network results in a trend towards a normal distribution of SPOR values. Though the SPOR has been designed to score pairwise collaboration, the graph database structure facilitates identification of high-scoring groups of providers through graph search methods (See Fig 4).

This study demonstrates that a healthcare collaboration network and the relationships it represents can be structurally evaluated to gain insight into the interactions that occur between healthcare providers in a hospital setting. Measuring collaborative relationships by risk-adjusted outcomes provides a relevant and informative basis for identifying successful patient care teams in medicine. Though we initially anticipated more variation in the SPOR value distribution from our test data, our results are not necessarily surprising and in fact have a positive connotation. The structure of the collaboration network makes it difficult for a collaborative relationship score to fall in either extreme. The small number of extreme values in this Emergency Department set suggests that the patient care environment in this hospital is consistent and stable.

This study has a number of limitations related to patient encounter data and outcome sources. First, since our test data set was extracted from electronic health records, the model cannot capture events such as cell phone and hallway conversations, text messages, personal conflicts, and other confounding factors that could potentially affect working relationships within a hospital environment. Second, the model has been tested using data from one domain (emergency medicine), and analysis with data sets from other hospital departments would provide an informative comparison. Third, even though we used a risk adjustment strategy, there may be other factors such as time of patient admission and daily provider caseload that could affect patient encounter outcomes. Finally, we chose patient satisfaction as the outcome in this study because it is considered an important factor in hospital quality measurement [70, 71]. However, there are potential drawbacks to basing outcomes on the Press Ganey survey. Depending on the time between the encounter and survey, a patient’s ability to remember events accurately may vary, leading to potential recall bias [72]. The bias is mitigated slightly by only surveying patients who are discharged immediately following their Emergency Department encounter in an effort to eliminate the confounding experience of an inpatient hospital stay. Because only non-admitted patients receive the survey, there tends to be a selection bias toward patients in less severe condition (i.e., lower acuity) [73]. Another factor contributing to non-randomness of this data is that patients receive only one satisfaction survey every 90 days regardless of the number of emergency room visits during that period. Also, patients who leave the emergency room without being evaluated by a provider will not participate in the survey [74]. Whether satisfaction indicates quality patient care has also been questioned [75]. While these factors are problematic, we believe that the significance of patient satisfaction for hospital reimbursement and comparison provided motivation for its use in demonstrating our method. Subsequent studies will explore other important outcomes such as 30-day hospital readmission [76, 77].

Future work will also address a variety of issues to improve our network model. First, we plan to modify the SPOR to measure collaboration between teams of 3 or more members. Information from additional sources will be added to enrich the network and improve its modeling capability. Further enrichment and expansion of the model will provide more effective recommendations for new care team members. Second, by including inpatient, outpatient, or other facility data in addition to ED records, collaborations within and between environments could be characterized and compared. Third, while the current study considers all activities to constitute equal contributions to encounter participation, it is easy to imagine that certain activities could contribute more or less to the quality of a patient’s hospital experience. For example, some procedures, while necessary, may cause pain or discomfort. Other activities may involve long wait times. Patients receiving these activities may be more inclined to be dissatisfied with their experience. In this study we chose to treat all activities equally, lacking definitive evidence to justify weighting any specific group of activities. However, because of the richness of our graph data model, such evidence, if available, could be employed to create a more specialized collaboration network. Studies to come will include a closer inspection of activity types and provider roles and their relationship to the SPOR. Through further analysis of specific protocols and related activities, it would be possible to find areas of excellence in collaboration as well as areas that can be targeted for improvement.

## Supporting Information

S1 File **Fig A, A Toy Example of a Provider-encounter Network.** Thirty nodes and fifty-three relationships are shown in this example network. Provider nodes (blue) are labeled with IDs. Encounter nodes are labeled with risk-adjusted outcome values. Each provider is linked to one or more encounters with an “INVOLVED_IN” relationship. The SPOR metric answers the following question: How many more good outcomes do two providers achieve when they work together versus when they work with any other provider? Therefore, in the situation where two providers collaborate exclusively with each other (regardless of how many other providers are involved), the SPOR score for their collaboration is 1. In relation to the provider-encounter network, these providers are usually in highly connected subgraphs, or cliques (P011, P012, P013, P014, and P015). The exception in this example is ‘P005, P004’, but these providers still share all of their respective encounters with each other. These collaborations are highlighted in red in Table A. The orange highlighted collaborations are between providers who share one encounter between them and one encounter with various other providers, which also results in a SPOR score of 1. **Table A, Toy Example SPOR values**. SPOR values for the provider collaborations in Fig A. **Table B, SPOR: Observed vs. Random**. An example of SPOR data for each collaboration and comparisons with the same collaboration score from permuted networks (those with collaborations based on randomized outcomes). Collaborations with a p-value ≤ 0.05 (high-scoring) or p-value ≥ 0.95 (low-scoring) were considered significant (example in bold). **Fig B, SPOR Statistics for Five Collaboration Networks**. Plot of descriptive statistics for the SPOR values of five collaboration networks, each with an increasing threshold for the number of shared encounters between providers. **Fig C, Provider Collaboration Network**. Nodes = providers, edges = collaborative relationships. The network included 574 providers and 5,615 relationships. An edge between two provider nodes indicates that they shared at least six patient encounters in our data set. Six modularity classes of providers were identified: Module 1 (light blue): 35.2%; Module 2 (dark blue): 17.5%; Module 3 (sea green): 16.5%; Module 4 (magenta): 15.6%; Module 5 (red): 12.2%; Module 6 (olive green): 1.7%. Nodes are sized according to degree centrality value, i.e., the number of others with whom a provider collaborated. **Table C, High-scoring SPOR group**. Twenty nine providers who had at least 5% of their total collaborative relationships in the highest 5% of SPOR scores (p-value ≤ 0.05). Fifteen providers (in blue) are present in both the top 5% and the bottom 5% (see Table D). The number of associated encounters with positive outcomes and the total number of associated encounters for each provider are shown in grey. **Table D, Low-scoring SPOR group**. Thirty eight providers who had at least 5% of their total collaborative relationships in the lowest 5% of SPOR scores (p-value ≥ 95%). Fifteen providers (in blue) are present in both the top 5% (see Table C) and the bottom 5%. The number of associated encounters with positive outcomes and the total number of associated encounters for each provider are shown in grey. **Table E, Encounter-level Summary Statistics by Acuity**. For the encounters in our data set, the mean and (median) number of providers and the length of stay (LoS) generally increased in accordance with the acuity value assigned to the patient upon arrival to the emergency department. Approximately 1% of the ED population is assigned acuity level “1 –Resuscitation”. The majority of these patients are admitted to the hospital due to the severity of their condition. These admitted patients do not receive the ED patient satisfaction survey, leading to the low number of encounters associated with this acuity level in our data set. **Fig D, Patient Age by Gender**. Our data set contained 4,477 female and 2,345 male patients. The mean (median) age for females was 50 (51). For males, mean (median) age was 53 (54). Due to its close proximity to a pediatric emergency department, the NMH ED accepts only patients who are 18+ years of age.
(DOCX)
