# Supplementary material for: An Outcome-Weighted Network Model for Characterizing Collaboration
Source: PLoS One. 2016 Oct 5;11(10):e0163861. doi: 10.1371/journal.pone.0163861 (PMC5051930; doi:10.1371/journal.pone.0163861)
Supplement: S1 File — Fig A, A Toy Example of a Provider-encounter Network. Thirty nodes and fifty-three relationships are shown in this example network. Provider nodes (blue) are labeled with IDs. Encounter nodes are labeled with risk-adjusted outcome values. Each provider is linked to one or more encounters with an “INVOLVED_IN” relationship. The SPOR metric answers the following question: How many more good outcomes do two providers achieve when they work together versus when they work with any other provider? Therefore, in the situation where two providers collaborate exclusively with each other (regardless of how many other providers are involved), the SPOR score for their collaboration is 1. In relation to the provider-encounter network, these providers are usually in highly connected subgraphs, or cliques (P011, P012, P013, P014, and P015). The exception in this example is ‘P005, P004’, but these providers still share all of their respective encounters with each other. These collaborations are highlighted in red in Table A. The orange highlighted collaborations are between providers who share one encounter between them and one encounter with various other providers, which also results in a SPOR score of 1. Table A, Toy Example SPOR values. SPOR values for the provider collaborations in Fig A. Table B, SPOR: Observed vs. Random. An example of SPOR data for each collaboration and comparisons with the same collaboration score from permuted networks (those with collaborations based on randomized outcomes). Collaborations with a p-value ≤ 0.05 (high-scoring) or p-value ≥ 0.95 (low-scoring) were considered significant (example in bold). Fig B, SPOR Statistics for Five Collaboration Networks. Plot of descriptive statistics for the SPOR values of five collaboration networks, each with an increasing threshold for the number of shared encounters between providers. Fig C, Provider Collaboration Network. Nodes = providers, edges = collaborative relationships. The network included 574 provider [file pone.0163861.s001.docx]

# Supporting Information


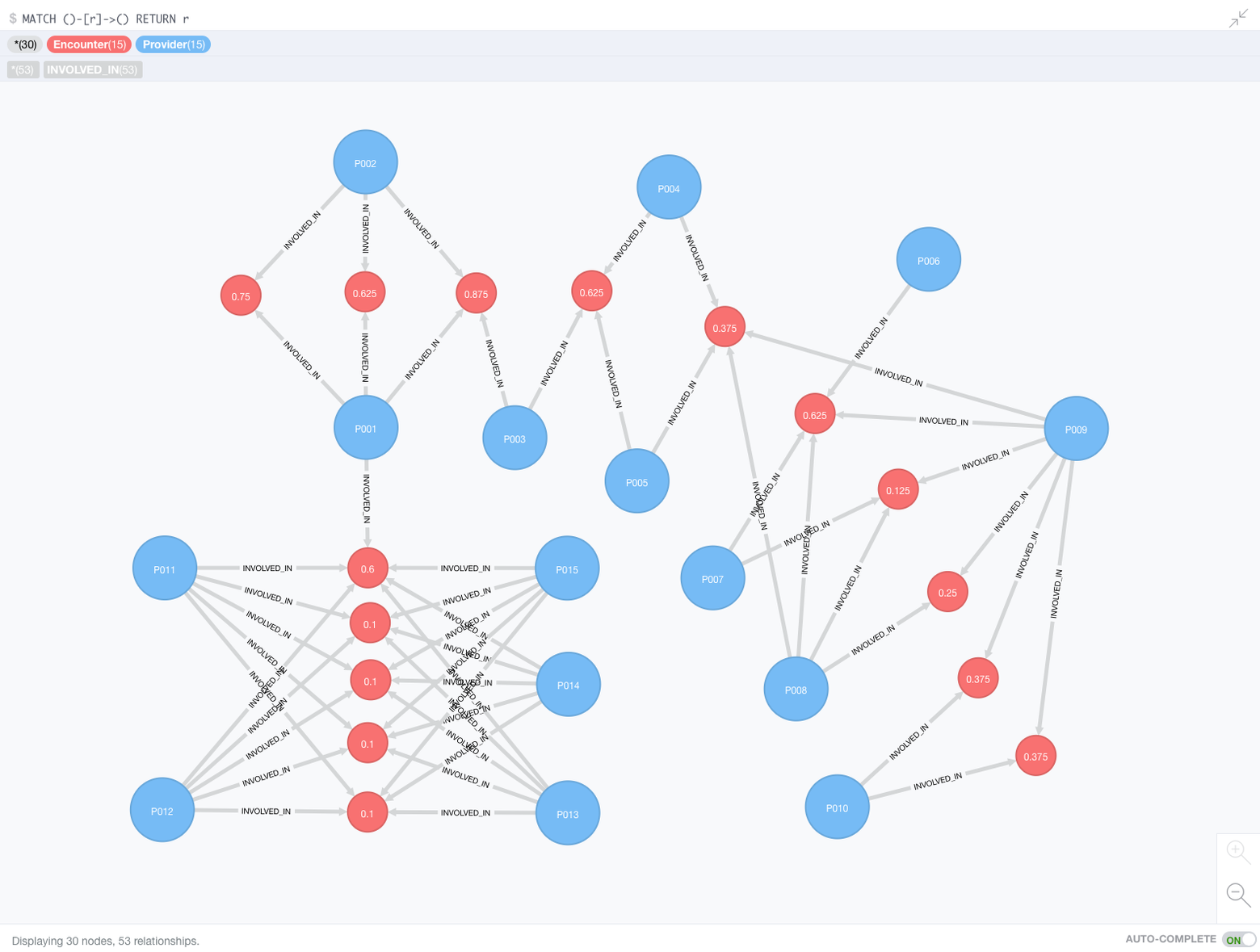


Fig A. A Toy Example of a Provider-encounter Network. Thirty nodes and fifty-three relationships are shown in this example network. Provider nodes (blue) are labeled with IDs. Encounter nodes are labeled with risk-adjusted outcome values. Each provider is linked to one or more encounters with an “INVOLVED_IN” relationship. The SPOR metric answers the following question: How many more good outcomes do two providers achieve when they work together versus when they work with any other provider? Therefore, in the situation where two providers collaborate exclusively with each other (regardless of how many other providers are involved), the SPOR score for their collaboration is 1. In relation to the provider-encounter network, these providers are usually in highly connected subgraphs, or cliques (P011, P012, P013, P014, and P015). The exception in this example is ‘P005, P004’, but these providers still share all of their respective encounters with each other. These collaborations are highlighted in red in Table A. The orange highlighted collaborations are between providers who share one encounter between them and one encounter with various other providers, which also results in a SPOR score of 1.

Table A. Toy Example SPOR Values.

SPOR values for the provider collaborations in Fig A.

Table B. SPOR: Observed vs. Random.

|  | ***Perm_0001_*** | ***Perm_0002_*** | ***Perm_0003_*** | ***…*** | ***Perm_x_*** | ***Obs*** | ***P-val*** |
| --- | --- | --- | --- | --- | --- | --- | --- |
| ***SPOR_12_*** | 0.984 | 1.30 | 1.15 | … | 1.07 | 1.09 | 0.21 |
| ***SPOR_13_*** | **1.09** | **1.05** | **0.782** | **…** | **1.11** | **1.37** | **0.01** |
| ***SPOR_22_*** | 0.996 | 0.913 | 1.03 | … | 0.859 | 1.04 | 0.35 |
| **…** | … | … | ... | … | … | … | … |
| ***SPOR_mn_*** | 1.17 | 1.12 | 0.73 | … | 1.01 | 1.01 | 0.41 |

An example of SPOR data for each collaboration and comparisons with the same collaboration score from permuted networks (those with collaborations based on randomized outcomes). Collaborations with a p-value ≤ 0.05 (high-scoring) or p-value ≥ 0.95 (low-scoring) were considered significant (example in bold).


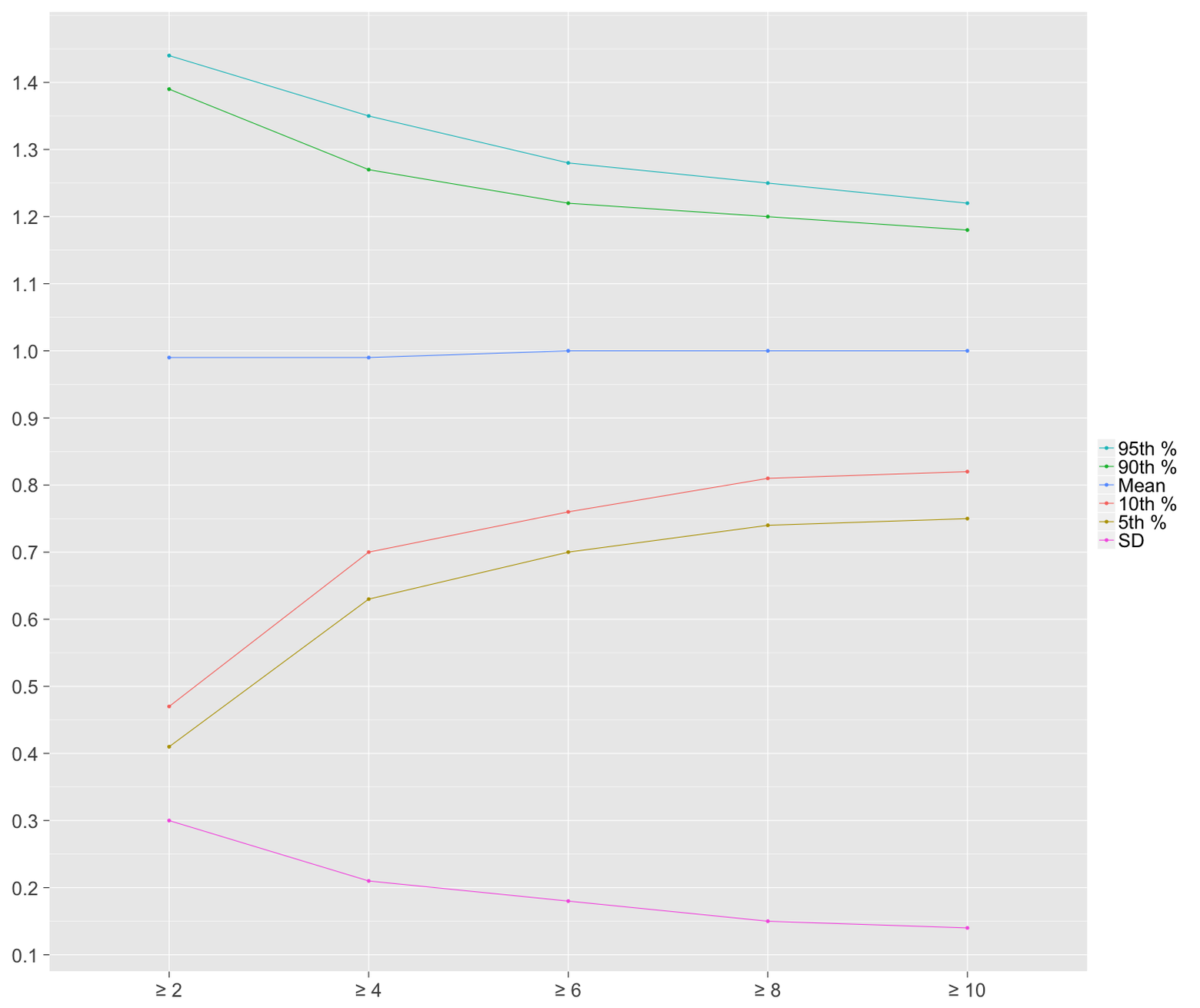


Fig B. SPOR Statistics for Five Collaboration Networks. Plot of descriptive statistics for the SPOR values of five collaboration networks, each with an increasing threshold for the number of shared encounters between providers.


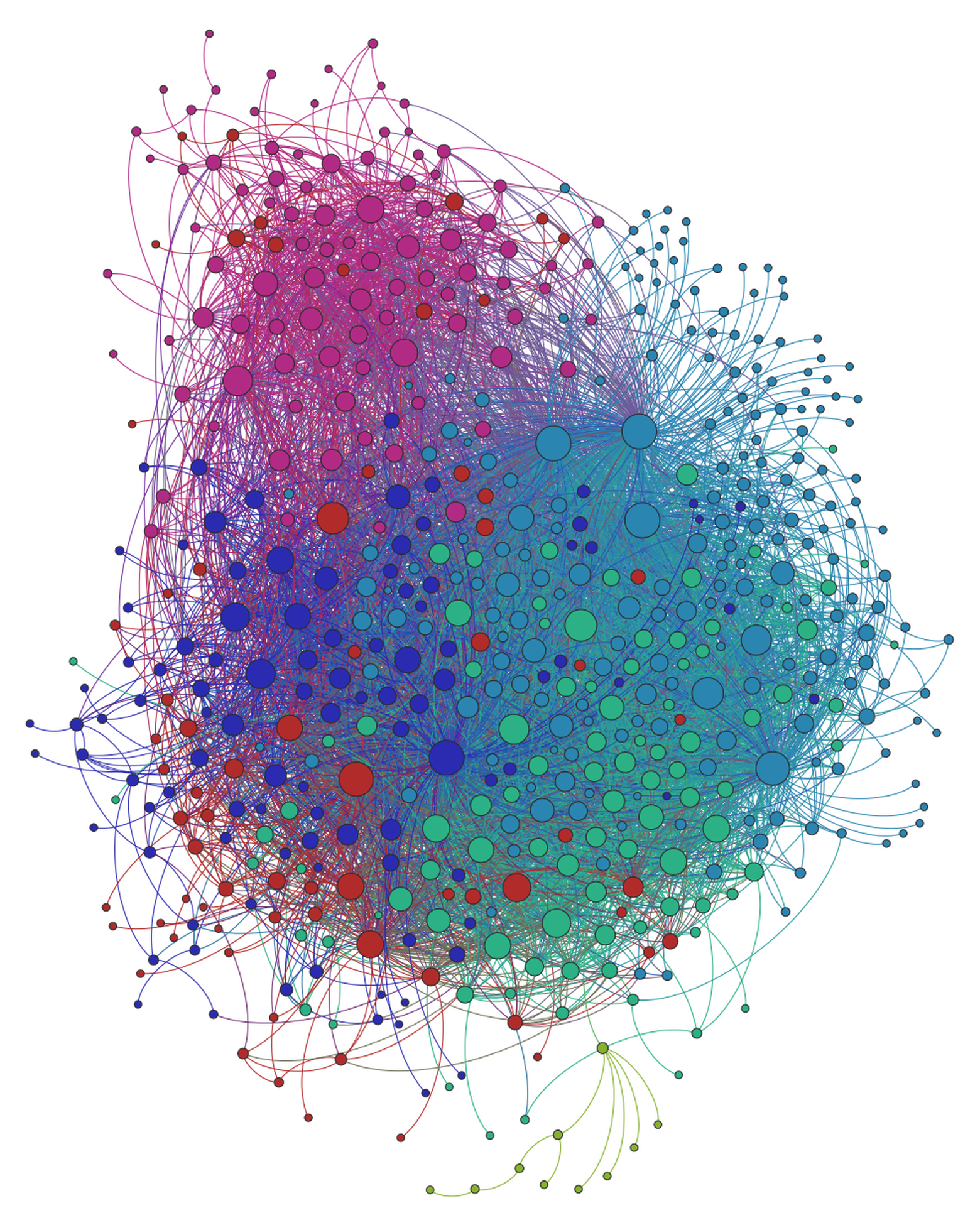


Fig C. Provider Collaboration Network. Nodes = providers, edges = collaborative relationships. The network included 574 providers and 5,615 relationships. An edge between two provider nodes indicates that they shared at least six patient encounters in our data set. Six modularity classes of providers were identified: Module 1 (light blue): 35.2%; Module 2 (dark blue): 17.5%; Module 3 (sea green): 16.5%; Module 4 (magenta): 15.6%; Module 5 (red): 12.2%; Module 6 (olive green): 1.7%. Nodes are sized according to degree centrality value, i.e., the number of others with whom a provider collaborated.

Table C. High-scoring SPOR Group.

| **High SPORs** | | | | | | | |
| --- | --- | --- | --- | --- | --- | --- | --- |
| **Provider Info** | | **Collaboration Stats** | | | **Associated Encounter Stats** | | |
| **DEID** | **Position** | **# SPORs p-val**  ≤ **0.05** | **# collabs** ≥ **6 encounters** | **%** | **Pos Outcomes** | **Total Encounters** | **% Encounters w/ Pos Outcome** |
| 9000578 | Radiologist | 17 | 311 | 5% | 333 | 523 | 64% |
| 9000465 | Radiologist | 14 | 293 | 5% | 330 | 513 | 64% |
| 9000728 | Radiology Tech | 15 | 281 | 5% | 249 | 411 | 61% |
| 9000120 | Radiologist | 15 | 266 | 6% | 270 | 435 | 62% |
| 9001032 | Medical Tech | 11 | 226 | 5% | 196 | 332 | 59% |
| 9000062 | Radiologist | 13 | 201 | 6% | 248 | 387 | 64% |
| 9000325 | ED Attending | 12 | 158 | 8% | 268 | 410 | 65% |
| 9001341 | Medical Tech | 8 | 151 | 5% | 135 | 242 | 56% |
| 9000343 | Physician Referral | 10 | 150 | 7% | 179 | 290 | 62% |
| 9000165 | Nurse | 5 | 126 | 4% | 170 | 312 | 54% |
| 9001699 | Medical Tech | 10 | 120 | 8% | 90 | 201 | 45% |
| 9001059 | Medical Tech | 6 | 115 | 5% | 115 | 205 | 56% |
| 9000385 | Medical Tech | 7 | 106 | 7% | 105 | 202 | 52% |
| 9000867 | Pharmacist | 5 | 105 | 5% | 115 | 194 | 59% |
| 9002700 | Laboratory Tech | 5 | 101 | 5% | 136 | 205 | 66% |
| 9000915 | Nurse | 7 | 90 | 8% | 152 | 251 | 61% |
| 9000320 | ED Attending | 8 | 88 | 9% | 166 | 263 | 63% |
| 9001036 | Medical Tech | 7 | 86 | 8% | 112 | 171 | 65% |
| 9000456 | Nurse | 5 | 84 | 6% | 151 | 229 | 66% |
| 9000679 | Nurse | 5 | 68 | 7% | 154 | 234 | 66% |
| 9000587 | Nurse | 5 | 61 | 8% | 111 | 223 | 50% |
| 9000018 | ED Attending | 6 | 60 | 10% | 137 | 199 | 69% |
| 9002390 | Medical Tech | 5 | 54 | 9% | 63 | 119 | 53% |
| 9000665 | Nurse | 6 | 50 | 12% | 114 | 169 | 67% |
| 9000859 | Laboratory Tech | 6 | 40 | 15% | 77 | 113 | 68% |
| 9001692 | Phlebotomist | 5 | 40 | 13% | 60 | 111 | 54% |
| 9000089 | Nurse | 5 | 37 | 14% | 108 | 164 | 66% |
| 9000163 | Nurse | 5 | 34 | 15% | 102 | 155 | 66% |
| 9002685 | Nurse | 5 | 22 | 23% | 77 | 125 | 62% |
|  |  |  |  |  |  |  |  |
|  | **Averages** | **8.03** | **121.52** | **6.6% (weighted)** | **155.97** | **254.76** | **61%**  **(weighted)** |

Twenty nine providers who have at least 5% of their total collaborative relationships in the highest 5% of SPOR scores (p-value ≤ 0.05). Fifteen providers (in blue) are present in both the top 5% and the bottom 5% (Table D). The number of associated encounters with positive outcomes and the total number of associated encounters for each provider are shown in grey.

Table D. Low-scoring SPOR Group.

| **Low SPORs** | | | | | | | |
| --- | --- | --- | --- | --- | --- | --- | --- |
| **Provider Info** | | **Collaboration Stats** | | | **Associated Encounter Stats** | | |
| **DEID** | **Position** | **# SPORs p-val**  ≥ **0.95** | **# collabs** ≥ **6 encounters** | **%** | **Pos Outcomes** | **Total Encounters** | **% Encounters w/ Pos Outcome** |
| 9000578 | Radiologist | 20 | 311 | 6% | 333 | 523 | 64% |
| 9000465 | Radiologist | 15 | 293 | 5% | 330 | 513 | 64% |
| 9000728 | Radiology Tech | 15 | 281 | 5% | 249 | 411 | 61% |
| 9000120 | Radiologist | 13 | 266 | 5% | 270 | 435 | 62% |
| 9001032 | Medical Tech | 10 | 226 | 4% | 196 | 332 | 59% |
| 9000062 | Radiologist | 5 | 201 | 2% | 248 | 387 | 64% |
| 9000325 | ED Attending | 11 | 158 | 7% | 268 | 410 | 65% |
| 9001341 | Medical Tech | 8 | 151 | 5% | 135 | 242 | 56% |
| 9000165 | Nurse | 9 | 126 | 7% | 170 | 312 | 54% |
| 9001699 | Medical Tech | 15 | 120 | 13% | 90 | 201 | 45% |
| 9001059 | Medical Tech | 11 | 115 | 10% | 115 | 205 | 56% |
| 9000385 | Medical Tech | 9 | 106 | 8% | 105 | 202 | 52% |
| 9000867 | Pharmacist | 5 | 105 | 5% | 115 | 194 | 59% |
| 9002068 | ED Attending | 9 | 94 | 10% | 107 | 230 | 47% |
| 9001043 | Lab Coordinator | 6 | 92 | 7% | 100 | 195 | 51% |
| 9000320 | ED Attending | 5 | 88 | 6% | 166 | 263 | 63% |
| 9002532 | Medical Tech | 10 | 88 | 11% | 80 | 156 | 51% |
| 9000480 | ED Attending | 7 | 80 | 9% | 140 | 227 | 62% |
| 9001713 | Medical Tech | 5 | 78 | 6% | 92 | 173 | 53% |
| 9000065 | ED Attending | 6 | 75 | 8% | 142 | 227 | 63% |
| 9001181 | Physician Referral | 8 | 71 | 11% | 123 | 201 | 61% |
| 9000146 | Nurse | 7 | 67 | 10% | 121 | 210 | 58% |
| 9002390 | Medical Tech | 6 | 54 | 11% | 63 | 119 | 53% |
| 9001728 | Nurse | 10 | 53 | 19% | 83 | 166 | 50% |
| 9001653 | Medical Tech | 5 | 48 | 10% | 75 | 128 | 59% |
| 9000026 | Radiologist | 5 | 47 | 11% | 54 | 112 | 48% |
| 9000396 | ED Attending | 10 | 45 | 22% | 92 | 171 | 54% |
| 9000806 | Radiology Tech | 6 | 45 | 13% | 57 | 128 | 45% |
| 9000390 | ED Attending | 10 | 44 | 23% | 74 | 156 | 47% |
| 9001196 | Nurse | 6 | 42 | 14% | 82 | 168 | 49% |
| 9001394 | Resident/Fellow | 6 | 42 | 14% | 102 | 163 | 63% |
| 9001775 | Lab Coordinator | 5 | 30 | 17% | 67 | 118 | 57% |
| 9000095 | Nurse | 6 | 29 | 21% | 63 | 121 | 52% |
| 9000181 | Radiology Tech | 10 | 29 | 34% | 38 | 87 | 44% |
| 9000794 | Nurse | 5 | 27 | 19% | 78 | 141 | 55% |
| 9000310 | Nurse | 5 | 26 | 19% | 54 | 139 | 39% |
| 9001065 | Medical Tech | 5 | 25 | 20% | 57 | 99 | 58% |
| 9002718 | Laboratory Tech | 5 | 23 | 22% | 55 | 90 | 61% |
|  |  |  |  |  |  |  |  |
|  | **Averages** | **8.26** | **100.03** | **8.26%**  **(weighted)** | **126.03** | **219.87** | **57%**  **(weighted)** |

Thirty eight providers who have at least 5% of their total collaborative relationships in the lowest 5% of SPOR scores (p-value ≥ 95%). Fifteen providers (in blue) are present in both the top 5% (see Table C) and the bottom 5%. The number of associated encounters with positive outcomes and the total number of associated encounters for each provider are shown in grey.

Table E. Encounter-level Summary Statistics by Acuity.

| **Acuity** | **# of Encounters** | **Provider Count** | **LoS** |
| --- | --- | --- | --- |
| **1 – Resuscitation** | 4 | 11 (10) | 5.1 (4.9) |
| **2 – Emergent** | 1705 | 11.1 (11) | 5.3 (4.6) |
| **3 – Urgent** | 3158 | 9.6 (9) | 4.9 (4.5) |
| **4 – Semi-urgent** | 1887 | 6.9 (7) | 3.3 (2.8) |
| **5 – Non-urgent** | 68 | 4.0 (4) | 1.7 (1.2) |

For the encounters in our data set, the mean and (median) number of providers and the length of stay (LoS) generally increased in accordance with the acuity value assigned to the patient upon arrival to the emergency department. Approximately 1% of the ED population is assigned acuity level “1 – Resuscitation”. The majority of these patients are admitted to the hospital due to the severity of their condition. These admitted patients do not receive the ED patient satisfaction survey, leading to the low number of encounters associated with this acuity level in our data set.


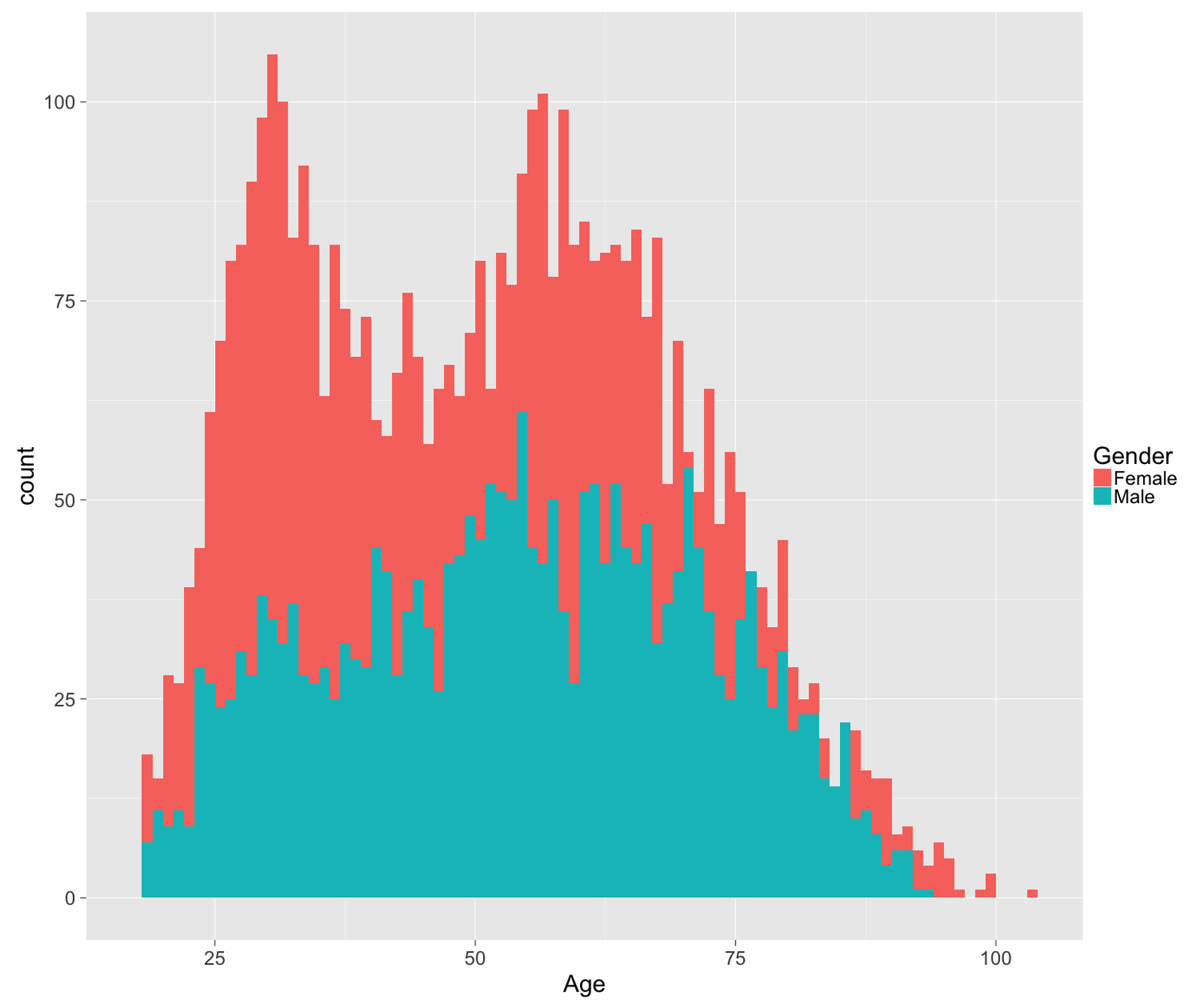


Fig D. Patient Age by Gender. Our data set contained 4,477 female and 2,345 male patients. The mean (median) age for females was 50 (51). For males, mean (median) age was 53 (54). Due to its close proximity to a pediatric emergency department, the NMH ED accepts only patients who are 18+ years of age.
